# An anti-cancer WxxxE-containing azurin polypeptide inhibits Rac1-dependent STAT3 and ERK/GSK-3β signaling in breast cancer cells

**DOI:** 10.18632/oncotarget.17759

**Published:** 2017-05-10

**Authors:** Zhe Zhang, Zhiyong Luo, Wenpu Min, Lin Zhang, Yaqun Wu, Xiaopeng Hu

**Affiliations:** ^1^ Department of Breast and Thyroid Surgery, Division of General Surgery, Tongji Hospital, Tongji Medical College, Huazhong University of Science and Technology, Wuhan, People's Republic of China; ^2^ The First People's Hospital of Jingzhou, Jingzhou, People's Republic of China

**Keywords:** WxxxE motif, azurin protein, Rac1, anti-cancer polypeptide, breast cancer

## Abstract

In our previous study, we characterized a mycoplasmal small GTPase-like polypeptide of 240 amino acids that possesses an N-terminal WVLGE sequence. The N-terminal WVLGE sequence promotes activation of Rac1 and subsequent host cancer cell proliferation. To investigate the function of the WxxxE motif in the interaction with Rac1 and host tumor progression, we synthesized a 35-amino acid WVLGE-containing polypeptide derived from a cell-penetrating peptide derived from the azurin protein. We verified that the WVLGE-containing polypeptide targeted MCF-7 cells rather than MCF-10A cells. However, the WVLGE-containing polypeptide inhibited activation of Rac1 and induced cellular phenotypes that resulted from inhibition of Rac1. In addition, the WVLGE-containing polypeptide down-regulated phosphorylation of the STAT3 and ERK/GSK-3β signaling pathways, and this effect was abolished by either stimulation or inhibition of Rac1 activity. We also found that the WVLGE-containing polypeptide has a Rac1-dependent potential to suppress breast cancer growth *in vitro* and *in vivo*. We suggest that by acting as a Rac1 inhibitor, this novel polypeptide may be useful for the treatment of breast cancer.

## INTRODUCTION

Earlier studies on the relation between infectious pathogens and host cells have established that WxxxE-containing bacterial proteins can activate host small GTPases that influence a wide range of cellular functions [1–3]. In our previous study, we characterized a mycoplasma-derived highly conserved 240 amino acid small GTPase-like polypeptide (SGLP) that enhances phosphorylation of the signal transducer and activator of transcription 3 (STAT3) by activation of Rac1 and therefore might promote tumor growth [4]. Because the SGLP-induced effects depend on the WxxxE motif in the conserved N-terminal domain of SGLP [4], we investigated the effect of the WxxxE motif per se rather than the full length of SGLP on Rac1 activity.

Rac1 is a pleiotropic small GTPase of the Rho superfamily that is involved in a diverse array of key cellular events, including cytoskeletal reorganization and cell proliferation [5–9]. As an important molecular switch that changes between GDP/GTP-bound forms, the active GTP-bound Rac1 interferes with many signal transduction molecules. Rac1 is correlated with STAT family members that stimulate breast cancer progression [10, 11]. Rac1 directly binds STAT3 and promotes STAT3 activation [12]. On the other hand, Rac1indirectly links integrin/FAK/PI3K signaling with the PRL-R/JAK2/STAT5 signaling pathway in mammary epithelial cells [13]. Besides activity in the receptor tyrosine kinase signal pathway, Rac1 promotes crosstalk with the Ras/Raf/MAPK pathway through ERK [14–16], which is an important serine/threonine kinase that inhibits breast cancer progression [17, 18]. The phosphorylation of serine 9 (S9) of GSK-3β was activated by ERK through its downstream molecule p90Rsk [19, 20], and inhibition of ERK might downregulate phosphorylation of GSK-3β (S9), which results in activation of GSK-3β [21–23]. The active GSK-3β promotes proteasome-dependent degradation of β-catenin [24–26], which downregulates the canonical Wnt/β-catenin pathway [27]. Therefore, we tested the possibility that the WxxxE motif of mycoplasma origin suppresses tumor growth through downregulation of STAT and ERK/GSK-3β signaling by inhibiting Rac1.

However, a practical method must be established to efficiently transport the WxxxE peptide into cancer cells. Azurin, a bacterial redox protein found in Pseudomonas aeruginosa, is a protein that has an anti-cancer effect as well [28–30]. Azurin contains a 28 amino acid cell-penetrating peptide (CPP), which makes it an ideal vehicle for transporting a covalently linked peptide sequence into cancer cells [31, 32].

We synthesized a 35 amino acid fusion polypeptide derived from the mycoplasmal WxxxE motif and azurin CPP, which can elicit an anti-cancer effect in breast cancer cells. We demonstrated that the underlying mechanism might be WxxxE-induced inhibition of Rac1 and subsequent suppression of STAT3 and ERK/GSK-3β/β-catenin signaling.

## RESULTS

### WVLGE-containing polypeptide correlates with small GTPase Rac1

**Figure 1:**
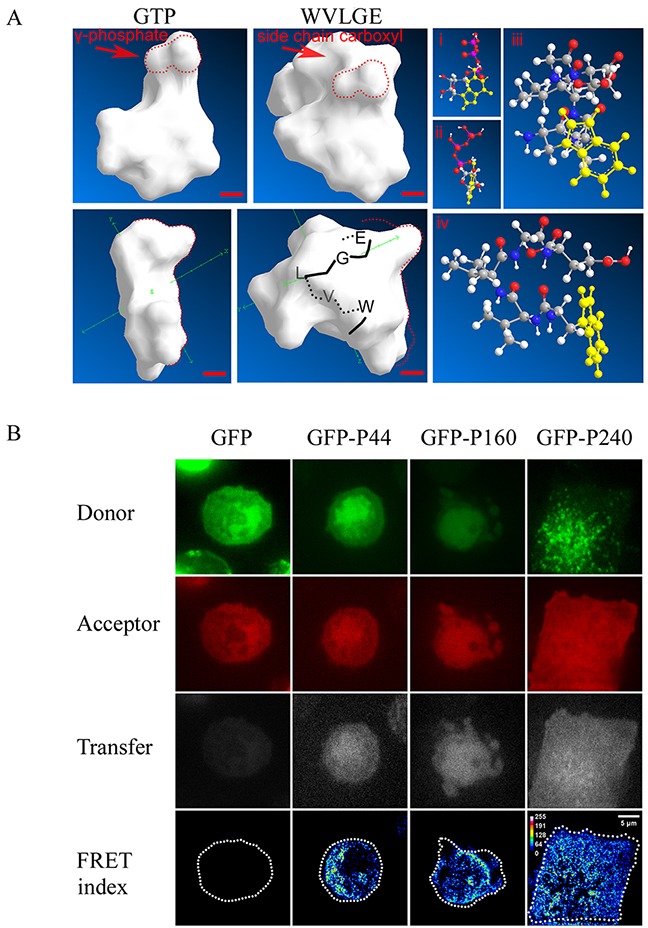
WVLGE-containing polypeptides derived from mycoplasma may directly interact with small GTPase Rac1 **(A)** Structural comparison between GTP molecule and WVLGE peptide. Front and side view of Connolly surface of the GTP molecule and the α-helical WVLGE peptide are presented (left panel), the red dotted line delineates the region of high similarity between γ-phosphate of GTP and carboxyl side chain of glutamic acid residue (‘E’). Scale bar represents 0.2 nm. The ball-and-stick models of GTP (i and ii) and WVLGE peptide (iii and iv) are presented as well (right panel). Each atom is indicated by the sphere's color as follows: white for hydrogen, black-gray for carbon, blue for nitrogen, and red for oxygen. The heterocyclic side chain of tryptophan (‘W’) and guanine part of the GTP molecule is highlighted in yellow. **(B)** HeLa cells co-expressing DsRed-Rac1 with GFP-tagged SGLP (GFP-P240), GFP-tagged N-terminal 160 amino acids of SGLP (GFP-P160), or GFP-tagged N-terminal 44 amino acids (GFP-P44) of SGLP were fixed in 4% paraformaldehyde and observed by confocal microscopy. Fluorescence resonance energy transfer (FRET) between DsRed-Rac1 (red) and GFP-P44, GFP-P160, or GFP-P240 were acquired at filter channels for donor, acceptor, and transfer respectively, and the FRET indices are plotted as pseudo-color images. Scale bar is 5 μm. Representative images from three independent experiments are presented.

In our previous study, we determined that exogenous mycoplasmal WVLGE-containing SGLP might be a factor in the activation of Rac1 [4]. In terms of structure, the indole ring of tryptophan in WVLGE is similar to the purine ring in GTP. Therefore, we investigated whether a structural resemblance exists between the WxxxE motif and GTP. As reported, the Connolly surface algorithm is a combination of van der Waals surface and solvent surface, generating a smooth surface contour that can be utilized in software to predict ligand-binding sites [33]. Thus, we simulated the molecular surface by use of ChemBioOffice software. The result revealed a similarity between the molecular surfaces of GTP and the α-helical WVLGE sequence (Figure 1A). In addition, the conserved WVLGE sequence that promotes SGLP-induced activation of Rac1 resides in the N-terminal domain of SGLP. To determine the interaction between WVLGE-containing polypeptide and Rac1, we measured the fluorescence resonance energy transfer (FRET) between co-expressed DsRed-Rac1 and GFP-tagged truncated N-terminal sequences of SGLP. The result revealed that the FRET index was high in the presence of WVLGE and the apparent efficiency was irrelevant to the size of WVLGE-containing polypeptides (Figure 1B), which indicates that the WVLGE-containing peptide is the minimal determinant for interaction with Rac1.

### Synthesized polypeptide derived from cell penetrating peptide of azurin protein can be introduced into breast cancer cell lines

**Figure 2:**
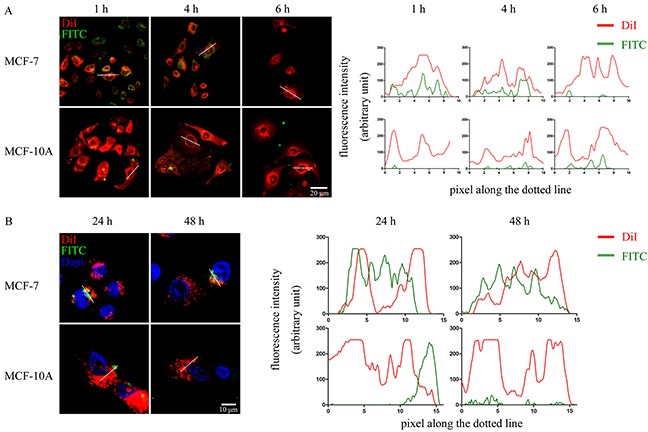
WVLGE-containing polypeptide enters into breast cancer cells **(A)** and **(B)** MCF-7 and MCF-10A cells were incubated with FITC-labeled WVLGE-containing azurin CPP for 24 hours and stained by membrane dye DiI for 15 minutes. Cells were incubated in fresh medium for different times as indicated before observation by confocal microscopy. Dapi was used to stain the nuclear for observation at 24 hours and 48 hours **(B)**. Scale bars are 20 μm **(A)** and 10 μm **(B)**, respectively. The fluorescence intensities of each pixel along the white dotted line are plotted. The X-axis represents the pixels along the white dotted line and the Y-axis represents the fluorescence intensity.

For inserting the WxxxE motif into cancer cells, the azurin CPP, serving as the vehicle for peptide transduction, was linked upstream to the WVLGE sequence. We investigated whether this synthesized peptide enters breast cancer cell lines. The MCF-7 cells and the nontumorigenic breast cell line MCF-10A were incubated with the FITC-labeled WVLGE-containing polypeptide at a physiological concentration of 1.0 μM and observed by confocal microscopy at different time points. In addition, the membrane dye DiI was used to mark the cellular boundary. The result showed that the polypeptide binds with MCF-7 cells but not with MCF-10A cells (Figure 2A). In a long-term incubation with MCF-7 cells, the fluorescence of the polypeptide overlapped with the perinuclear DiI-positive vesicles, which was not observed in MCF-10A cells (Figure 2B).

### WxxxE polypeptide inhibits activation of Rac1 in breast cancer cells

**Figure 3:**
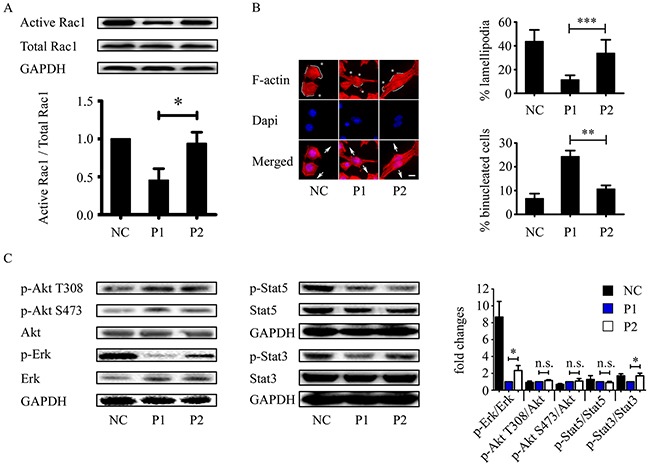
WVLGE-containing polypeptide inhibits Rac1 activity and Rac1-associated cellular phenotypic changes **(A)** MCF-7 cells were incubated with WVLGE-containing polypeptide (P1), VWLGE-containing polypeptide (P2) at 1 μM, or negative control (NC) for 24 hours before lysis. A GST-Pak1 pull-down assay was applied for detection of Rac1 activity. Representative data from three independent experiments are presented. The active Rac1 level of each group was normalized to NC and plotted as indicated (mean ± SD). **(B)** MCF-7 cells treated as in **(A)** were fixed and stained by Phalloidin-TRITC and Dapi before fluorescence microscopy. Asterisk indicates fillopodium, solid line along the cell periphery indicates lamellipodium, and arrow pairs indicate separating direction of cytokinesis. Scale bar is 10 μm. Percentage of lamellipodia and binucleated cells were plotted as indicated (n = 30, mean ± SD). **(C)** Rac1-associated phosphorylation of Akt, ERK, STAT3, and STAT5 were determined by Western blot analysis. The representative data from three independent experiments are presented. Fold changes of relative phosphorylation levels were normalized to each of P1 and plotted as indicated (mean ± SD). *P<0.05, **P<0.01, ***P<0.001, n.s.: not significant.

The hypothesis that the WxxxE motif disrupts host cellular small GTPase Rac1 led us to investigate the effect of the WxxxE polypeptide on the activation of Rac1. In an active Rac1 pull-down assay, we showed that active Rac1 level decreased in MCF-7 cells treated with WVLGE-containing polypeptide but remained nearly unchanged in point-mutated cells treated with the VWLGE-containing polypeptide compared with the control groups (Figure 3A).

Because inhibited Rac1 exhibits less lamellipodia formation and more prominent filopodia [34, 35], we investigated the cytoskeletal F-actin distribution patterns in MCF-7 cells treated with WVLGE-containing polypeptide, VWLGE-containing polypeptide, or negative control. The fluorescent phalloidin-staining of F-actin in MCF-7 cells treated with WVLGE-containing polypeptide exhibited a phenotype of Rac1 inhibition as compared with negative control and cells treated with VWLGE-containing polypeptide (Figure 3B), which correlates with the result of the active Rac1 pull-down assay that the WxxxE motif induces inhibition of Rac1. In addition, WVLGE-containing polypeptide increased binucleated cells, a phenomenon of cytokinesis failure associated with Rac1 activity [36, 37], in MCF-7 cells (Figure 3B). Because active Rac1 is with a factor in Akt, ERK, and STAT3/5 signaling [16, 38–40], we investigated the phosphorylation of Akt, ERK, and STAT3/5 by Western blot analysis and the results showed that p-ERK and p-STAT3 (Y705) levels decreased in MCF-7 cells treated with WVLGE-containing polypeptide as compared with cells treated with VWLGE-containing polypeptide (Figure 3C), which is consistent with the explanation that inhibition of Rac1 by WVLGE-containing polypeptide results in downregulation of ERK and STAT3 signaling.

### WxxxE-induced inhibition of phosphorylation of STAT3 (Y705), ERK, and GSK-3β (S9) is dependent on Rac1 activity

**Figure 4:**
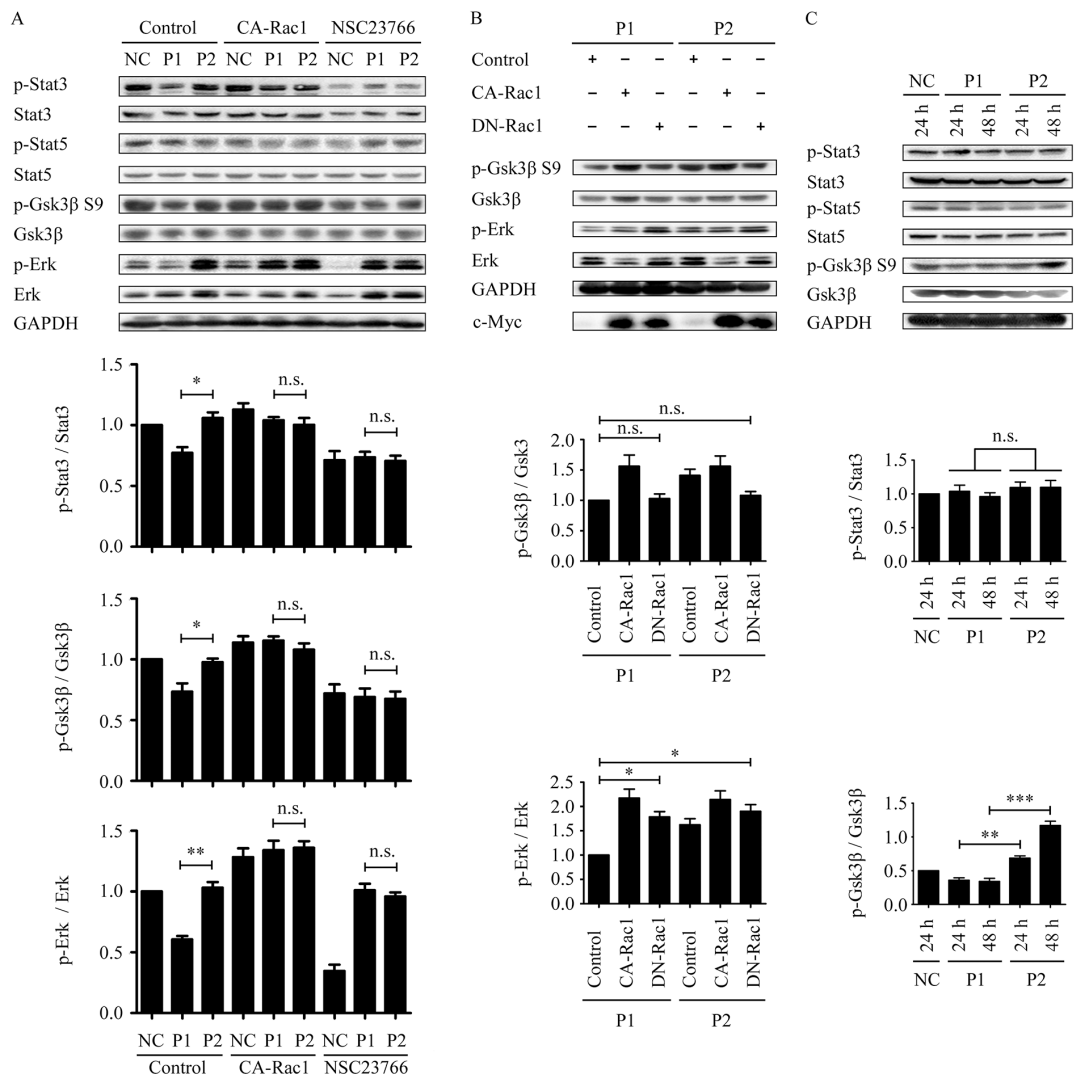
WVLGE-induced decrease in the phosphorylation of STAT3 and ERK/GSK-3β is Rac1-dependent, and the effect on ERK/GSK-3β is time-dependent **(A)** MCF-7 cells were treated as described in Supplementary Figure 1. The densitometric ratio of p-STAT3/STAT3, p-GSK-3β/GSK-3β, and p-ERK/ERK were normalized and plotted as indicated (n = 3, mean ± SEM). **(B)** MCF-7 cells were transfected with CA-Rac1 or DN-Rac1 for 24 hours followed by incubation with 1.0 μM WVLGE-containing polypeptide (P1) or VWLGE-containing polypeptide (P2) for another 24 hours before harvest. The densitometric ratio of p-GSK-3β/GSK-3β and p-ERK/ERK are normalized and plotted as indicated (n = 3, mean ± SEM). **(C)** MCF-7 cells were incubated with P1-polypeptide or P2-polypeptide at 100 nM for 24 hours or 48 hours, respectively, in addition to negative control for 24 hours. The densitometric ratio of p-STAT3/STAT3 and p-GSK-3β/GSK-3β are normalized and plotted as indicated (n = 3, mean ± SEM). *P<0.05, **P<0.01, ***P<0.001; n.s.: not significant. (NC: negative control; CA-Rac1: constitutive active Rac1; DN-Rac1: dominant negative Rac1; NSC23766: Rac1 inhibitor).

We investigated whether the WxxxE-induced effect on phosphorylation of STAT3 and ERK is dependent on Rac1 activity. The results revealed that WVLGE-containing polypeptide rather than VWLGE-containing polypeptide decreased p-STAT3 (Y705), and that the decrease is reversed by co-expression of constitutively active Rac1 (CA-Rac1) but is suppressed by the addition of the Rac1 inhibitor NSC23766 (Figure 4A), which was not observed for STAT5. The WVLGE-induced decrease in p-ERK level recapitulates the dependence on Rac1 activity (Figure 4A). In addition, the decrease in phosphorylation of GSK-3β serine 9 (S9), as a downstream event in ERK1/2 signaling [20, 23], is also dependent on Rac1 activity (Figure 4A).

NSC23766 treatment in combination with either WVLGE-containing polypeptide or VWLGE- containing polypeptide elicited an upregulation of p-ERK as compared with a WVLGE-treated negative control (Figure 4A), which obscures the underlying mechanism. To investigate whether the paradox is restricted to NSC23766, we studied the p-ERK and the p-GSK-3β (S9) levels in MCF-7 cells incubated with WVLGE-containing polypeptide or VWLGE-containing polypeptide co-expressing dominant negative Rac1 (DN-Rac1). The result confirmed that inhibition of Rac1 by the co-expression of DN-Rac1 in combination with either WVLGE-containing polypeptide or VWLGE-containing polypeptide also increased p-ERK level as compared with a control vector in combination with WVLGE-containing polypeptide (Figure 4B). The implication is that the relative increase in p-ERK level when Rac1 is inhibited is caused by the concomitantly added azurin CPPs, ascribed to the CPP backbone sequence rather than to the WxxxE motif.

We found that the WVLGE-induced effect on p-GSK-3β (S9) existed at a low peptide concentration as compared with that of p-STAT3, and that the divergence between p-GSK-3β (S9) levels of the MCF-7 cells treated with WVLGE-containing polypeptide and those treated with VWLGE-containing polypeptide become much more noticeable as incubation time increases (Figure 4C). The divergence indicates that the effect of the WxxxE motif on p-GSK-3β is time-dependent rather than dose-dependent.

### WxxxE-induced phosphorylation of β-catenin and decrease in total β-catenin level is Rac1-dependent

**Figure 5:**
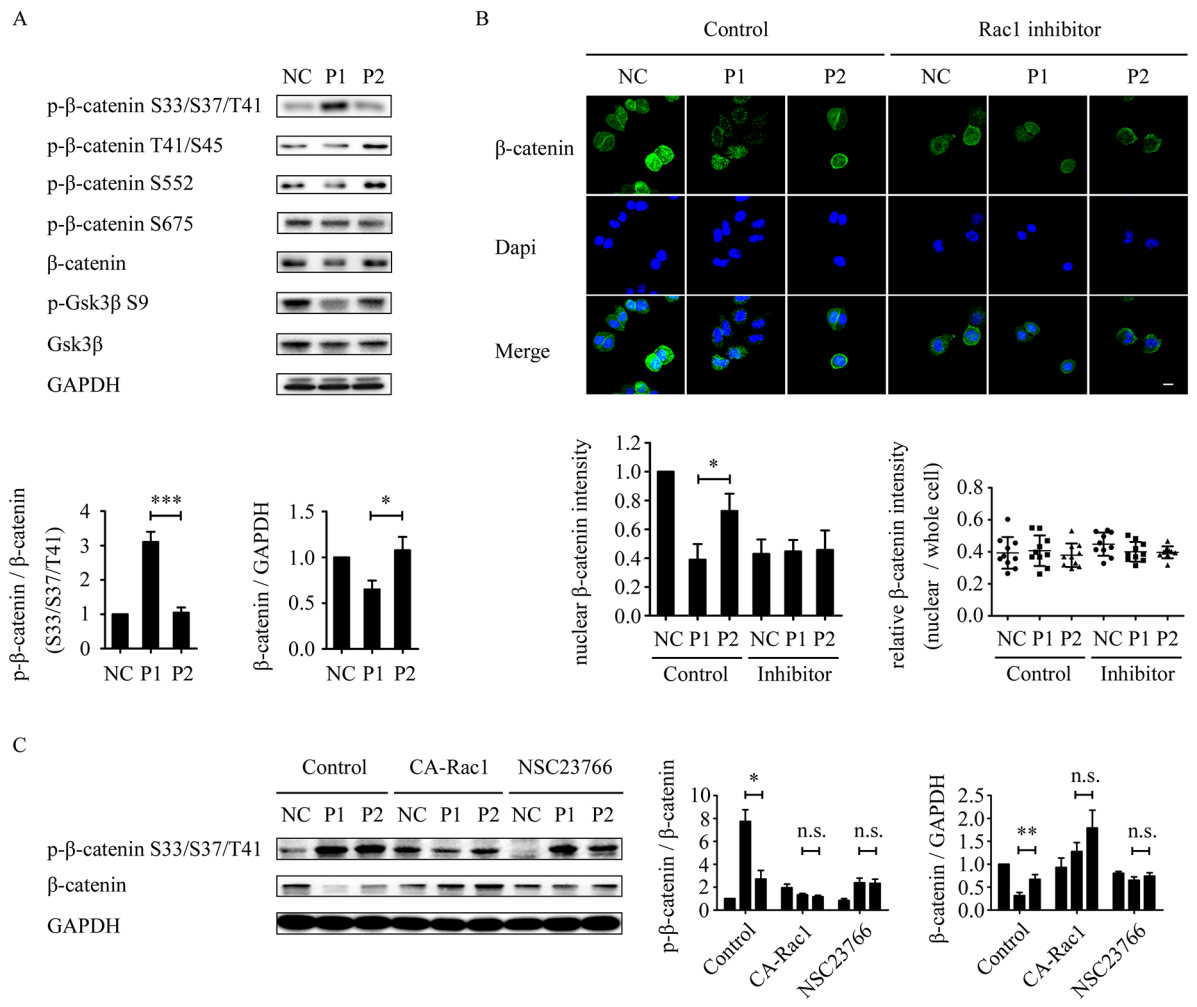
WVLGE-induced depletion of β-catenin is correlated with GSK-3β and Rac1-dependent **(A)** MCF-7 cells were incubated with WVLGE-containing polypeptide (P1), VWLGE-containing polypeptide (P2) at 1 μM, or negative control (NC) for 24 hours before lysis. The densitometric ratio of p-β-catenin/β-catenin and β-catenin/GAPDH were normalized and plotted as indicated (n = 3, mean ± SD). **(B)** MCF-7 cells were treated as in **(A)** followed by incubation in combination with or without NSC23766 (Rac1 inhibitor) for another 24 hours. The cells were immunostained for β-catenin and counterstained by Dapi before confocal microscopy. The fluorescence intensity of nuclear β-catenin was analyzed by ImageJ and plotted as indicated (n = 10, mean ± SD). The relative intensity of nuclear β-catenin to whole cells were analyzed by ImageJ and plotted as indicated (n = 10). **(C)** MCF-7 cells were treated as described in Supplementary Figure 1. The densitometric ratio of p-β-catenin/β-catenin and β-catenin/GAPDH were normalized and plotted as indicated (n = 3, mean ± SEM). *P<0.05, **P<0.01, ***P<0.001, n.s.: not significant. (CA-Rac1: constitutive active Rac1).

Because dephosphorylation of GSK-3β (S9) promotes phosphorylation of β-catenin at residues S33, S37, and T41 [24], leading to degradation of β-catenin by the ubiquitin-proteasome pathway [26], we investigated whether the phosphorylation of β-catenin (S33/S37/T41) is increased by WVLGE-containing azurin CPP. The result showed that the WVLGE-containing polypeptide increased phosphorylation of β-catenin (S33/S37/T41) and a decrease in the total β-catenin level (Figure 5A). In addition, several other phosphorylation sites of β-catenin, such as T41/S45, S552, and S675, corresponding to the targets of IKKα, Akt, and PKA, respectively [41–45], were also investigated. The result showed that phosphorylation level of those sites is not changed (Figure 5A). We investigated the intracellular distribution of β-catenin by immunofluorescence. The result showed that in MCF-7 cells treated with WVLGE-containing polypeptide, the nuclear β-catenin intensity decreased proportionally to the total β-catenin level, which is suppressed by Rac1 inhibitor NSC23766 (Figure 5B). We also investigated whether the WVLGE-induced increase in phosphorylation of β-catenin (S33/S37/T41) and the decrease in total β-catenin is dependent on Rac1 activity. The MCF-7 cells were treated as in Figure 4A, and p-β-catenin (S33/S37/T41) and total β-catenin level were studied by Western blot analysis. The result showed that WVLGE-containing polypeptide provoked phosphorylation of β-catenin (S33/S37/T41), accompanied by a decrease in total β-catenin level as compared with mutated VWLGE-containing polypeptide. Moreover, the different p-β-catenin (S33/S37/T41) and total β-catenin levels between MCF-7 cells treated with WVLGE-containing polypeptide and VWLGE-containing polypeptide is suppressed by either co-expression of CA-Rac1 or addition of NSC23766 (Figure 5C), which implies that the WVLGE-induced alteration in β-catenin is Rac1-dependent.

### Anticancer effect of WxxxE-containing polypeptide in breast cancer cells

**Figure 6:**
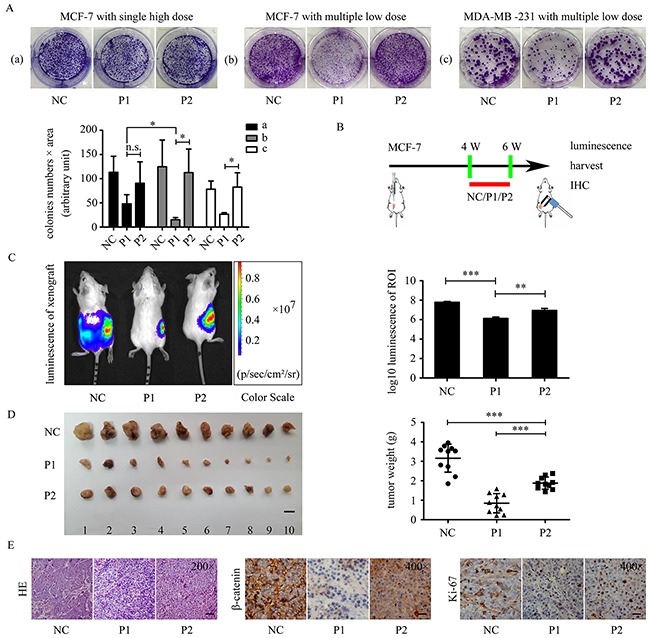
WVLGE-containing polypeptide suppresses growth of breast cancer cells **(A)** Colony formation assay in MCF-7 and MDA-MB-231 cells. ‘Single high dose’ stands for adding the polypeptides to 1.0 μM once at the beginning of incubation, and ‘multiple low dose’ stands for adding the polypeptides to 0.10 μM 5 times every 3 days. The number of cells in the colonies multiplied by the area of the colonies are plotted at the lower left panel (n = 3, mean ± SD). **(B)** The schematic graph shows the experimental design of the *in vivo* study. Four weeks after MCF-7 cells were transplanted, the tumor xenografts were treated with P1-polypeptide, P2-polypeptide, or negative control for 2 weeks, followed by observation. **(C)** Tumor xenografts of MCF-7 luc+ cells were treated as described in **(B)**. The bioluminescence of the tumor xenografts were recorded as pseudo-color images according to the color scale presented. Red dotted lines delineate the region of interest (ROI). The luminescence intensities of the ROI were quantified and normalized by log10 (n = 3, mean ± SD). **(D)** Tumor weights were plotted as indicated. Scale bar is 1 cm (n = 10, mean ± SD). **(E)** The representative images of HE staining and IHC of β-catenin and Ki-67. Scale bar is 100 μm for HE and 50 μm for IHC images. P1-treated tumor xenograft possesses fewer tumor parenchymal cells compared with P2 and negative control groups. IHC were performed on adjacent 5-μm sections for β-catenin and Ki-67. Quantitative data of IHC are provided in Supplementary Figure 2. *P<0.05, **P<0.01, and ***P<0.001; n.s.: not significant.

To evaluate the anticancer ability of WxxxE-containing polypeptide in breast cancer, we examined the effect of WVLGE-containing polypeptide on the proliferation of breast cancer cells *in vitro* and *in vivo*. Colony formation assay showed that the WVLGE-containing polypeptide suppressed the colony formation of MCF-7 and MDA-MB-231 cells compared with the negative control and point-mutated VWLGE-containing polypeptide groups (Figure 6A). Moreover, the result showed that the suppression effect of WVLGE-containing polypeptide on colony formation in MCF-7 cells is more prominent at multiple low doses than at a single high-dose, which indicates that the anti-proliferation effect of WVLGE-containing polypeptide is time-dependent rather than dose-dependent (Figure 5A). In breast tumor xenograft assays, MCF-7 cells stably transfected with the luciferase gene were injected subcutaneously near hind limbs of nude mice and treated as described in the schematic graph (Figure 6B), followed by bioluminescence imaging, tumor measurement, and immunohistochemistry (IHC). After treatment everyday with or without the WxxxE-containing polypeptide at a low concentration for 2 weeks, visualizations were conducted for differently treated groups. The luminescence images revealed that WVLGE-containing polypeptide suppressed the growth of the implanted tumors (Figure 6C). Quantitative analysis based on the luminescence intensity also revealed that WVLGE-containing polypeptide suppressed the growth of the implanted tumors as compared with the point-mutated VWLGE-containing polypeptide and negative control (Figure 6C). Tumor weight measurement showed that both the WVLGE-containing polypeptide and the VWLGE-containing polypeptide suppressed the growth of the implanted tumors compared with the negative control, and the WVLGE-polypeptide suppressed tumor growth much more than the VWLGE-containing polypeptide (Figure 6D). The paraffin-embedded tumors were processed for hematoxylin-eosin (HE) staining and IHC study for β-catenin and Ki-67. The HE staining showed that MCF-7 cells treated with WVLGE-containing polypeptide possess fewer parenchymal cells at the cancer foci, and IHC revealed that β-catenin expression level and numbers of Ki-67-positive cells decreased in the groups treated with WVLGE-containing polypeptide (Figure 6E).

## DISCUSSION

In several species, both animals and plants, the WxxxE motif in a variety of bacterial proteins exert bio-effects and cause disease through activation of small GTPases in host cells [2, 46]. This cross-kingdom activity indicates the persistence and importance of the WxxxE motif in evolution, thus making it valuable for research.

Our previous study demonstrated that mycoplasma-derived protein fragments of SGLP, which consists of 240 amino acid residues and an N-terminal WVLGE peptide sequence could promote phosphorylation of STAT3 by activating Rac1. Computer simulation showed structural homology between α-helical WVLGE and GTP molecules, which implies potential cross-activation and/or cross-inactivation of host small GTPases by exogenous WVLGE-containing polypeptides. We focused on the conservative N-terminal WVLGE-containing domain of SGLP to investigate whether the WVLGE motif is critical for interaction with Rac1. As predicted, the interaction with Rac1 is related to the WxxxE motif *per se* rather than to the length of surrounding sequences.

We linked the most simplified AWVLGEA sequence downstream to azurin-derived cell-penetrating peptide. Our data confirmed that this optimized 35 amino acid polypeptide can be selectively targeted into breast cancer cells. The WVLGE-containing polypeptide inhibited activation of Rac1 rather than promoted it. The WVLGE-containing polypeptide caused Rac1-associated phenotypic alterations, such as reduction of lamellipodia and increase in cytokinesis failure, which can be attributed to inhibiting Rac1 either locally or globally [37, 47]. However, the local inhibition of Rac1 at the cleavage furrow can rescue cytokinesis failure [37], which suggests that other cytokinesis-associated small GTPases, such as RhoA and cdc42 [48, 49], might promote a WVLGE-induced increase in binucleated cells. Although other small GTPases might be factors, inhibition of Rac1 can explain the current alterations observed in cells treated with WVLGE-containing polypeptide. We demonstrated that the phosphorylation of ERK and STAT3, downstream molecules correlated with Rac1 [16, 50, 51], was suppressed by WVLGE-containing polypeptide, which is the result of WVLGE-induced Rac1 inhibition. We further demonstrated that the discrepancy between the effect of WVLGE-containing polypeptide and VWLGE-containing polypeptide on p-STAT3, p-ERK, and p-GSK-3β can be eliminated either by expression of constitutively active Rac1 or by addition of Rac1 inhibitor NSC23766. This outcome indicates that WVLGE-induced downregulation of STAT3, ERK, and GSK-3β phosphorylation is dependent on Rac1 activity. Therefore, the exogenous WxxxE motif inhibits Rac1 activity and might downregulate the Rac1-associated signaling pathway.

Because dephosphorylation of GSK-3β (S9) is activated on β-catenin, we investigated the effect of WVLGE-containing polypeptide on the expression of β-catenin. The results showed an increase in phosphorylation of β-catenin at the S33, S37, and T41 sites accompanied by a decrease in the total β-catenin level. The WVLGE-containing polypeptide elicited a decrease in nuclear β-catenin, and the nuclear to whole-cell ratio of β-catenin in all groups was unchanged, which suggests that WVLGE-containing polypeptide promotes GSK-3β-activated β-catenin degradation without changing intracellular β-catenin distribution. We also demonstrated that the WVLGE-induced decrease in β-catenin is dependent on Rac1 activity. We propose that the WxxxE motif might downregulate the GSK-3β/β-catenin pathway through inhibition of Rac1 activity.

Colony formation assay exhibited a more prominent growth-inhibiting effect on breast cancer cell lines with multiple low doses than with a single high dose of WVLGE-containing polypeptide. This outcome suggests that the anti-cancer effect of WVLGE-containing polypeptide is time-dependent rather than dose-dependent. Thus, we applied multiple low-dose polypeptides in tumor xenografts experiments to investigate the *in vivo* anti-cancer effect of WVLGE-containing polypeptide. The growth of transplanted breast tumors is suppressed by WVLGE-containing polypeptide. Immunohistochemistry of transplanted tumors treated with WVLGE-containing polypeptides showed reduction of parenchymal cells in the cancer foci and lower expression of β-catenin and Ki-67. Alterations in these important pathological parameters suggest an anti-cancer effect of WVLGE-containing polypeptide.

Our findings revealed that the novel WVLGE-containing polypeptide might serve as a breast cancer therapeutic through inhibition of Rac1 and Rac1-stimulated signaling pathways. Although the mutant VWLGE-containing polypeptide has no effect on Rac1 activity, slight suppression of tumor growth by VWLGE-containing polypeptide occurs compared with WVLGE-containing polypeptide. This finding is consistent with a previous report that the azurin CPPs have anti-cancer effect [32]. Theoretically, the anti-tumor mechanism of the WxxxE motif might be different from that of azurin CPPs. However, these two peptide sequences might exert the anti-cancer effect synergistically. Thus, further efforts on the modification of the WxxxE-azurin fusion peptide without damaging its anti-cancer potency will be the focus of our future research.

## MATERIALS AND METHODS

### Molecular surface analysis

The molecular surface was drawn by the ChemBio3D module of ChemoBioOffice Ultra 2010 software (Cambridge Soft, MA, USA). The structure of the WVLGE peptide sequence was set as in an α-helix, and the Connolly surface algorithm was applied for surface simulation [33].

### Cell lines

HeLa, MCF-7, MCF-10A, and MDA-MB-231 cells were purchased from ATCC (Manassas, VA, USA) and used in early passes. HeLa cells were cultured in Dulbecco's Modified Eagle Medium (Hyclone, Logan, UT, USA) supplemented with 10% fetal bovine serum (Hyclone). MCF-7 cells were cultured in Eagle's Minimum Essential Medium (Hyclone) supplemented with 0.01 mg/mL of human recombinant insulin and 10% fetal bovine serum (Hyclone). MDA-MB-231 cells were cultured in L-15 medium supplemented with 10% fetal bovine serum. MCF-10A cells were cultured in MEBM (Hyclone) supplemented with 100 ng/mL cholera toxin and 10% fetal bovine serum. MCF-7 cells were stably transfected with the luciferase gene by adding commercial lentiviral particles (S&E Bio-Pharmaceutical Technology Co., Shanghai, China) containing pCMV-mCherry-2A-luciferase gene at a multiplicity of infection of 10. The MCF-7 luciferase positive (MCF-7 luc+) cells were selected and maintained as described previously [4]. All cells were maintained in a humidified 5% CO_2_ atmosphere at 37°C.

### Plasmids and transfection

Constitutively active Rac1 (CA-Rac1) and dominant negative Rac1 (DN-Rac1) plasmids were purchased from Addgene (Cambridge, MA, USA). Lipofectamine™ 2000 (Invitrogen Life Technologies, Carlsbad, CA, USA) was used for transfection of plasmids per the manufacturer's instruction. For each 10 cm^2^ well, 4 μg DNA and 10 μL Lipofectamine were diluted in 250 μL Gibco™ Opti-MEM (Invitrogen) respectively and incubated at room temperature for 5 minutes before mixture. The 500 μL mixture were incubated at room temperature for 20 minutes before adding to the 2 mL Opti-MEM at 90-95% cell confluence. Fresh medium was changed after 6 hours.

### Fluorescence resonance energy transfer

MCF-7 cells were plated on glass-bottomed petri dishes and fixed in 4% paraformaldehyde before confocal microscopy. Images were acquired by use of an Olympus confocal microscope with a 100×/1.40 oil lens. FRET was acquired at three channels for donor, acceptor, and transfer, and analyzed by ImageJ software FRET and Colocalization Analyzer plugin. The donor and acceptor Bleed Through was determined and the apparent FRET index was plotted as described [52].

### Polypeptide and reagent

Polypeptide (GeneScript China, Nanjing, Jiangsu, China) was synthesized by use of FlexPeptide^TM^ technology. The sequences are LSTAADMQGVVTDGMASGLDKDYLKPDDAWVLGEA (P1) and LSTAADMQGVVTDGMASGLDKDYLKPDDAVWLGEA (P2). FITC conjugated P1 was provided by GeneScript China as well. Polypeptide powder was resolved in PBS to a final concentration of 1.0 μM before use and PBS served as control. NSC23766 (Selleck Chemicals, Houston, TX, USA) was resolved in H_2_O and used at a final concentration of 100 μM in cell cultures.

### Immunofluorescent staining and fluorescence microscopy

MCF-10A and MCF7 cells were treated with FITC conjugated P1-polyptptide (GeneScript Biotechnology) for 24 hours before DiI (Beyotime Biotechnology, Shanghai, China) staining for 15 minutes. Cells were then cultured in fresh medium for 1, 4, 6, 24, or 48 hours and observed by an Olympus FV1000 confocal microscope, for which Dapi (Beyotime) of 0.3 mM was used for nuclear counterstaining in 24-hour and 48-hour groups.

MCF-7 cells were treated with 1.0 μM of P1-polypeptide, P2-polypeptide (GeneScript), or control for 24 hours, and then cells were fixed and permeabilized before staining with 50 μg/mL Phalloidin-TRITC (Sigma) at room temperature for 2 hours. Cells were then counterstained with Dapi and observed by use of an Olympus BX51 wide field microscope. The cell perimeter occupied by lamellipodia was defined as an actin-rich fringe as described [53]. The percentage of lamellipodia is measured by ImageJ. The binucleated cells are defined as cells that contain two nuclei and determined by two uninformed observers.

MCF-7 cells were treated with P1-polypeptide, P2-polypeptide (1.0 μM), or control for 24 hours, followed by incubation with or without NSC23766, and then cells were fixed in 4% paraformaldehyde for 30 minutes, rinsed with PBS twice, and permeabilized with 0.1% Triton-X100 in PBS for 10 minutes. Cells on coverslips were blocked with 5% BSA in PBS for 30 minutes, and then incubated with anti-β-catenin (Cell Signaling Technology) at 4°C overnight. Cells were washed three times and incubated with goat-anti-rabbit Alexa 488-conjugated secondary antibodies (Invitrogen, California, USA) for 30 minutes at 37°C before counterstaining with Dapi for 5 minutes. Confocal images were acquired by use of an Olympus FV1000 microscope. Images were processed by ImageJ software for presentation.

### Western blot analysis

Active Rac1 pull-down assay and Western blot analysis were performed as previously described [4]. MCF7 Cells incubated with polypeptides at 1.0 μM were lysed in a NP-40 lysis buffer (0.15 M NaCl, 1% NP-40, and 0.05 M Tris-HCl, pH 8.0) with a mixture of protease inhibitors (0.25 mM phenylmethylsulfonyl fluoride, 10 mg/mL aprotinin and leupeptin, and 1 mM dithiothreitol). Approximately 20 μg of total protein was separated via 10% SDS-PAGE and transferred to a PVDF membrane (Millipore, MA, USA). The primary antibodies purchased from Cell Signaling Technology were as follows: anti-p-Akt (Thr308, #13038), anti-p-Akt (Ser473, #4060), anti-Akt (#4691), anti-p-ERK (Thr202/Tyr204, #4094), anti-ERK(#4695), anti-p-STAT5 (Tyr694, #4322), anti-p-STAT3 (Tyr705, #4113), anti-STAT5(#94205), anti-p-GSK-3β (Ser9, #9323), anti-GSK-3β (#12456), anti-p-β-catenin (Ser33/37/Thr41, #9561), anti-p-β-catenin (Thr41/Ser45, #9561), anti-p-β-catenin (Ser552, #5651), anti-p-β-catenin (Ser675, #4176), anti-β-catenin (#8480), and anti-GAPDH (#5174). The primary antibodies purchased from Santa Cruz Biotechnology were anti-STAT3 (SC-8019) and anti-c-Myc (SC-40).

### Colony formation assay

MCF-7 and MDA-MB-231 cells were plated in a six-well plate at a density of 5×10^2^ cells per well and incubated with P1-polypeptide, P2-polypeptide, or negative control for 2 weeks followed by staining with crystal violet. Colony forming ability was calculated as the number of cells in colonies multiplied by the area of discrete colonies obtained by particle function of ImageJ.

### Tumor xenografts assay

Female BALB/c nu/nu mice at 4 weeks of age were purchased from the Institute of Laboratory Animal Science, Chinese Academy of Medical Sciences (Beijing, China). At 8 weeks post-natal, (sexually mature) mice were injected with 1 × 10^6^ MCF-7 luc+ cells mixed with 50 μL extracellular matrix (BD Transduction Laboratories, Franklin Lakes, NJ, USA) subcutaneously into fat pads near the hind limbs. Four weeks after injection, when implanted tumors began to appear, the mice were divided into three groups and treated with P1-polypeptide, P2-polypeptide (each time, 50 nmol/site) or PBS control injection in situ in 2-day interval for 2 weeks. The mice with tumor xenografts were anesthetized and injected intravenously (caudal vein) with 150 mg/kg D-luciferin (Xenogen, Berkeley, MA, USA) before being photographed by use of an *in vivo* bioluminescence imaging system (Xenogen, Berkeley, CA, USA). Then tumors were removed from mice and the tumor weight was recorded immediately. IHC was performed as previously described [54, 55].

### Statistical analysis

Data plotting and statistical analysis were conducted using GraphPad Prism 5.0 software (GraphPad Software, Inc., La Jolla, CA, USA). Two-tailed unpaired Student's *t*-tests were used for comparisons between groups, and paired *t*-tests were used for comparisons between normalized data groups in Figure 3C and Figure 4B.

## SUPPLEMENTARY FIGURES


