# Supplementary material for: An anti-cancer WxxxE-containing azurin polypeptide inhibits Rac1-dependent STAT3 and ERK/GSK-3β signaling in breast cancer cells
Source: Oncotarget. 2017 May 10;8(26):43091–103. doi: 10.18632/oncotarget.17759 (PMC5522130; doi:10.18632/oncotarget.17759)
Supplement: Supplementary file 1 [file oncotarget-08-43091-s001.pdf]

An anti-cancer WxxxE-containing azurin polypeptide inhibits Rac1-dependent STAT3 and ERK/GSK-3β signaling in breast cancer cells

SUPPLEMENTARY FIGURES

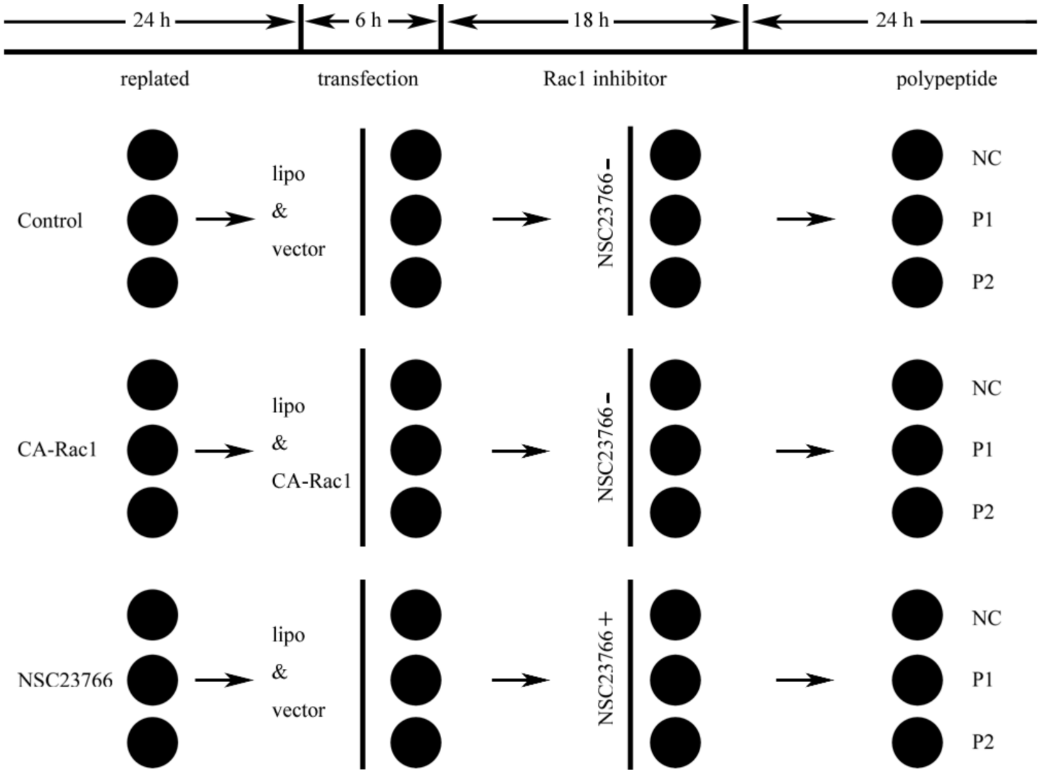

Supplementary Figure 1: The MCF-7 cells underwent treatment described as follows: replated in dishes (day 1), transfed with CA-Rac1 or control plasmids (the first 6 h, day 2), incubated with or without Rac1 inhibitor NSC23766 (the last 18 h, day 2), incubated with or without 1.0 μM polypeptides before harvest (day 3).

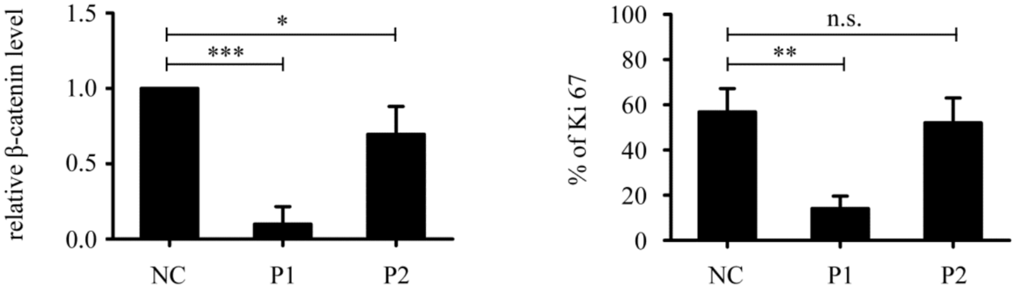

Supplementary Figure 2: Quantitative analysis of IHC for β-catenin and Ki-67. Tumor xenografts were treated by WVLGE-(P1), VWLGE-polypeptide (P2) or negative control (NC). Expression level of β-catenin (left, normalized to NC, n = 5, mean ± SD) and percentage of Ki-67 positive cells (right, n = 3, mean ± SD) were measured by Image-Pro Plus. *, $P<0.05$, **, $P<0.01$ and ***, $P<0.001$; n.s.: not significant.
